# The need for a cultural shift from two-arm to multi-arm RCTS

**DOI:** 10.1186/1745-6215-14-S1-O3

**Published:** 2013-11-29

**Authors:** Matthew R Sydes, Mahesh KB Parmar

**Affiliations:** 1 MRC Clinical Trials Unit, London, UK; 2 MRC Clinical Trials Unit Hub for Trials Methodology Research, London, UK

## Proposal

We need a faster and more efficient way of improving patient outcomes in all disease areas. There is a need for a cultural shift in randomised phase III superiority trials. We propose there be fewer two-arm trials and more trials that incorporate multiple research arms.

## Setting

Well-designed, well-conducted randomised controlled trials (RCTs) are the most reliable way to collect comparative evidence on efficacy and effectiveness, and drive changes in policy and healthcare. A literature search has shown that ~80% of all trials have only 2 arms. A key problem is their low success rate, defined as a convincing finding that the research treatment is better than the current standard-of-care. New treatments commonly do not work as well as hoped, yet we persist with traditional phase III RCTs, that are both expensive and often take many years. A direct conclusion from the high failure rate is that the information on which phase III trials are designed must be flawed. In planning any 2-arm phase III trial, fundamental uncertainties often remain; for example, whether we have reliably selected the most appropriate dose and/or duration of treatment or, for the academic community, whether other treatments have equal (or greater) priority for being assessed?

## Conclusion

An academic RCT should not be seen as a way of assessing a specific treatment, but considered as a central tool to improving outcomes for patients with a particular condition as quickly as possible. Therefore, we argue that many more trials should have more than one research arm.

